# Protocol for a systematic review and meta-analysis on the associations between short rest periods between shifts and health, function, and behavioral outcomes

**DOI:** 10.1186/s13643-026-03146-5

**Published:** 2026-03-06

**Authors:** Filip Borgen Andersen, Ståle Pallesen, Ingvild Thorsen Egaas, Øystein Vedaa, Morten Birkeland Nielsen, Erlend Sunde, Øystein Holmelid, Bjarte Birkeland Kysnes, Bjørn Bjorvatn, Siri Waage, Annina Ropponen, Anne Helene Garde, Anna Dahlgren, Ingebjørg Louise Rockwell Djupedal, Anette Harris

**Affiliations:** 1 Department of Psychosocial Science, University of Bergen, Bergen, Norway; 2 Norwegian Competence Center for Sleep Disorders, Haukeland University Hospital, Bergen, Norway; 3 Department of Health Promotion, Norwegian Institute of Public Health, Bergen, Norway; 4 Department of Work Psychology and Physiology, National Institute of Occupational Health, Oslo, Norway; 5 Department of Global Public Health and Primary Care, University of Bergen, Bergen, Norway; 6 The Finnish Institute of Occupational Health, Helsinki, Finland; 7 The National Research Centre for the Working Environment, Copenhagen, Denmark; 8 Division of Insurance Medicine, CNS, The Karolinska Institutet, Stockholm, Sweden; 9 Department of Research Administrative Support, Norwegian Institute of Public Health, Bergen, Norway; 10 Department of Psychology, Stockholm University, Stockholm, Sweden; 11 The Division of Psychology, Department of Clinical Neuroscience, Karolinska Institutet, Stockholm, Sweden

**Keywords:** Shift work, Quick returns, Between-shift breaks, Work performance, Social factors, Work-related factors

## Abstract

**Purpose:**

This paper presents a protocol for a planned systematic review and meta-analysis that will investigate the impact of short rest periods between shifts (i.e., quick returns, <11 h rest) on various outcomes in terms of physical health of workers (e.g., cardiovascular disease, infections, headaches, back pain) and/or mental health of workers (e.g., anxiety, depression, fatigue, sleep problems, insomnia, impaired mental health), as well as sickness absence (proxy of health), work performance (e.g., occupational accidents), social factors (e.g., social support), and psychosocial work-related factors (e.g., job satisfaction). The planned review further aims to identify different types of quick returns, such as evening to day shift and night to evening shift, and examine their relationship to these outcomes provided a sufficient number of studies (e.g., ≥5). The research will identify demographic and methodological factors that may moderate the relationship between quick returns and the specified outcomes.

**Methods:**

A systematic review and meta-analysis will be carried out. Predefined search terms for short rest between consecutive shifts will be developed and employed to identify relevant studies examining quick returns and all aforementioned outcomes. The databases MEDLINE, Web of Science, PsychInfo, EMBASE, ProQuest, BASE, and Cochrane Trials and the 200 first hits in Google Scholar will be searched up to April 2026. Two reviewers will independently screen identified records and assess eligible full texts for inclusions. The risk-of-bias of included studies will be assessed using the Mixed Methods Appraisal Tool (MMAT) for systematic mixed study reviews. A random effects meta-analytic synthesis will be conducted using Comprehensive Meta-Analysis software. The primary measure of effect size will be odds ratio with 95% confidence interval. Heterogeneity will be evaluated using Cochran’s* Q* test and the *I*^2^ statistics. Publication bias will be assessed using Duval and Tweedie trim and fill procedure. The protocol adheres to PRISMA and MOOSE reporting guidelines and is registered in the International Prospective Register of Systematic Reviews.

**Discussion:**

This planned systematic review and meta-analysis will be the first to synthesize the current evidence systematically and quantitatively regarding the consequences of quick returns, including demographic and methodological moderators. Aggregation of the existing evidence will improve our understanding of the consequences of quick returns and, as such, provide directions for practitioners in terms of possible preventive initiatives and work environment improvements.

**Systematic review registration:**

PROSPERO CRD42024533607.

**Supplementary Information:**

The online version contains supplementary material available at 10.1186/s13643-026-03146-5.

## Introduction

Shift work refers to any work schedule that takes place outside the hours of 0700–1800 h and may include evening, night, and early morning shifts as well as fixed or rotating schedules [1]. The growing trend of shift work and irregular working hours is widely attributed to significant societal changes, particularly the decline in manufacturing and the rise of the service sector [2]. In situations where shift workers are assigned to rotate between different shifts, rest periods of <11 h between two consecutive shifts may occur; this is denoted a “quick return.” This arrangement is commonly observed when an evening shift is followed by a morning shift, but it may occur between all types of shifts [3, 4]. Quick returns allow workers to compress their work weeks, providing them with longer periods of days off. However, quick returns have also been associated with insufficient recovery and an increased risk for injury [5]. The European Union’s working time directive (2003/88/EC, Article 3) therefore emphasizes that member states should ensure that workers have a minimum of 11 h of daily rest per 24 h [4]. Nevertheless, 23% of European workers report experiencing at least one episode of quick return within the last month [6].

Quick returns have been linked to a range of adverse effects [3, 7–10]. The most notable impact is the limited duration of sleep, which in a systematic review was found to be as short as 5.5 to 6.5 h compared to the 7.0 to 8.0 h of sleep when workers are not working quick returns [3]. Insufficient sleep is believed to be the primary underlying mechanism for the negative health consequences associated with shift work in general [11, 12] such as an increased risk of metabolic disorders, cardiovascular diseases, cancer, dementia, and depressive symptoms [13–16].

In addition to inadequate sleep duration, observational and register studies have found an association between working quick returns and an increased risk of poor sleep quality, insomnia, and excessive sleepiness and fatigue [3, 7–10]. However, the findings on other potential outcomes associated with quick returns are mixed and less consistent. For example, working quick returns has been associated with an increased risk of sickness absence among Norwegian and Finnish workers, but not among Danish workers [17–19]. Similarly, inconsistent results have been reported for the association between quick returns and stress. While a cross-sectional study showed a positive association between quick returns and stress among medical doctors [20], a study on newly graduated nurses found no between-person effect of quick returns on stress [21]. Furthermore, quick returns could potentially influence lifestyle factors through its association with insomnia, stress, and fatigue [3]. However, a 6-year follow-up study found no association between quick returns and physical activity, caffeine intake, or alcohol and nicotine consumption [22]. More consistent findings have been reported for the association between quick returns and an increased risk for work-related accidents [5, 23, 24]. The inconsistencies in the literature may be attributed to differences in study populations and operationalization of central variables but other work time variables could also be important. For instance, those working quick returns may also be exposed to long shifts, long work weeks, etc. In addition, the inconsistencies may also be a result of the quality or risk-of-bias of the studies themselves. In summary, existing findings on the impact of quick returns on health, work performance, social factors, and psychosocial work-related factors are fragmented and inconclusive. Consequently, there is a need for a systematic synthesis of the evidence that can elucidate the potential outcomes of quick returns on workers.

To address this gap in the research field, this planned systematic review and meta-analytic study will provide a comprehensive examination of the literature on the effects of quick returns on the above-mentioned outcome categories. Specifically, the primary objectives are to determine if quick returns are associated with (i) the physical health of workers (e.g., cardiovascular disease, infections, headaches, back-pain) and/or the mental health of workers (e.g., anxiety, depression, fatigue, sleep problems, insomnia, impaired mental health), as well as sickness absence (proxy of health), (ii) their work performance (e.g., occurrence of occupational accidents), (iii) social factors (e.g., social support), and (iv) psychosocial work-related factors (e.g., job satisfaction), as well as identifying different types of quick returns (e.g., evening to morning/day shift and night to evening shifts) and their relationship with these outcomes. Additionally, the study aims to identify moderating factors, such as age, sex, occupational groups (healthcare workers versus not), actual resting time between shifts (continuous variable), instruments utilized (single item scales versus multi-item scales), and sample size, that may affect the relationship between quick returns, health, function, and behavioral outcomes. This research will build upon existing literature on quick returns and provide valuable insights into the development of health-promoting work schedules.

## Methods

The methodology employed in this protocol article adheres to the reporting guidelines specified in the Preferred Reporting Items for Systematic Review and Meta-Analysis Protocols (PRISMA-P) [25] and the reporting guidelines for Meta-Analyses and Systematic Reviews of Observational Studies (MOOSE) [26]. A completed PRISMA-P checklist is provided in Additional file 1. The protocol has been registered in PROSPERO with the registration number: CRD42024533607. The proposed systematic review and meta-analysis are part of the overarching project: “Towards a Sustainable Workforce in the Healthcare Sector for the 21st century: Health-promoting Work Schedules” [27]. The objective of Health-promoting Work Schedules is to establish a comprehensive understanding of health-promoting work schedules among healthcare workers and to facilitate the development of a productive and sustainable workforce for the future healthcare sector.

### Data sources and search strategy

Relevant articles for the review and meta-analysis will be identified through systematic searches in MEDLINE, Web of Science, PsychInfo, EMBASE, ProQuest, and Cochrane Trials. To include grey literature, searches will also be conducted in BASE (Bielefeld Academic Search Engine) and Google Scholar, with the first 200 hits being considered. Moreover, the reference lists of full-text articles will be scrutinized to identify any further eligible studies (backward citation search). The search approach will be tailored for each database included, incorporating the search terms specified in Table 1. The search results will be limited to articles that are written in a European language. No time frame constraints will be employed. The search will be conducted by consulting with a librarian, and the bibliographic software EndNote will be utilized to eliminate any duplicate entries. The principal investigator (AH) will oversee the entire search process.

**Table 1:** Example of a search string used in the literature search

((short adj (changeover or “change over” or rest or recovery or “off duty” or offduty or turnaround or “turn around” or “free time” or “time between shifts” or “breaks between shifts” or “shift interval” or “inter shift interval?” or “intershift interval?” or “inter shift break?” or “intershift break?”)) or (quick adj (return? or changeover or “change over” or changes or turnaround or “turn around” or “shift change period?” or “shiftchange period?”)) or ((“fast rotat*” or irregular or “rapidly rotat*” or “frequently rotat*”) adj shift?) or ((“inter shift” or intershift) adj (“rest periods <11 hours” or “rest periods <eleven hours” or “rest periods under 11 hours” or “rest periods under eleven hours” or “intervals of <11 hours” or “intervals of <eleven hours”)) or “11 hours or less between two consecutive shifts” or “eleven hours or less between two consecutive shifts” or (“shift? separated by less than” adj (“11 hours” or “eleven hours”)) or ((“inter shift” or intershift) adj (“rest periods <10 hours” or “rest periods <ten hours” or “rest periods under 10 hours” or “rest periods under ten hours” or “intervals of <10 hours” or “intervals of <ten hours”)) or “10 hours or less between two consecutive shifts” or “ten hours or less between two consecutive shifts” or (“shift? separated by less than” adj (“10 hours” or “ten hours”))).tw	

### Inclusion and exclusion criteria

#### Study type

Eligible studies will be limited to quantitative observational studies that utilize cross-sectional, prospective, or retrospective designs. Single-case studies and studies with qualitative designs will be excluded. Lastly, articles published in languages other than European languages, as well as conference abstracts, will be excluded.

#### Participants

Studies eligible for inclusion should report original findings on individuals who have been subjected to shift work, irregular working hours, or simulated shift work with at least one instance of short rest periods between two consecutive shifts (quick returns), defined as less than 11.0 h between two shifts. Eligible studies will consist of working participants between the ages of 18 and upwards. Studies involving on-call workers and participants under the age of 18 will be excluded.

#### Exposure

The exposure will consist of quick returns. Included studies must provide their respective zero-order associations (e.g., correlations and odds ratios between quick returns and specified outcomes) or sufficient information for effect sizes to be calculated. If a study lacks such information, the respective author will be contacted and asked to provide the data.

#### Comparators

For studies to be considered for inclusion, they have to report a viable comparator for groups exposed to quick returns, which is either day work or non-exposure to quick returns.

#### Outcomes

Included studies should report original findings on the relationships between quick returns and all health-related outcomes, including the physical health of workers (e.g., cardiovascular disease, infections, headaches, back pain) and/or mental health of workers (e.g., anxiety, depression, fatigue, sleep problems, insomnia, impaired mental health), as well as sickness absence (proxy of health), and performance outcomes such as cognitive function and occupational accidents. Additionally, social-related factors, such as social relation, work-family balance, and social support, and psychosocial work-related factors, such as job satisfaction and turnover intention, will be included, provided that they are reported with regard to their association with quick returns.

### Data management and selection process

Two reviewers, FBA and ITE, will assess studies identified during the literature searches based on the criteria outlined in Table 2. The Rayyan software will be utilized to structure the screening and assessment process [28]. During the initial screening, the search results will be evaluated based on their title and abstract. In the second stage of screening, full-text articles from the first screening will be assessed in terms of the applicable inclusion and exclusion criteria provided above. If the raters/evaluators disagree, they will discuss the matter until they reach consensus. If disagreements persist, a third independent researcher will be consulted to resolve the issue. This procedure is designed to minimize bias in the decision-making process and ensure correct inclusion or exclusion of studies. The screening and selection process will be illustrated using the PRISMA flowchart [29].

**Table 2:** Checklist for inclusion or exclusion of the review studies

Language and literature	Design	Exposure	Control group/comparator	Outcome	Data analysis	Results
Full text, and European language	Observational study, cross-sectional, prospective or retrospective, case-control, cohort, experimental	Quick returns: i.e., 11.0 h or less between two consecutive shifts	Non-quick returns/regular working hours: working hours with 11.0 h or more between shifts	Health, work performance, social factors, and psychosocial work-related factors	Analyses with comparison between quick returns and control groups	Results of comparison between quick returns and the control group (or provided by authors)

### Data extraction

The data will be extracted using a predefined and piloted data extraction/coding sheet, which will be independently applied by the two reviewers. Before its application, the coding sheet will be developed and pre-tested on relevant articles, with any necessary adjustments made. Training and discussions regarding coding will take place to ensure high inter-rater reliability and the correct application of variables in line with the sheet. This process is expected to improve the overall precision and congruence of coding. The following data will be extracted and coded: first author name and publication year, working country of the sample, data collection period, study design, occupational groups (nurses, doctors, soldiers, etc.), sample size (total, female, male), response rate, age of participants (range, mean and standard deviation), type of quick return (e.g., evening to morning shift, morning to night shift) assessment method (registry, self-report, etc.), instrument utilized to measure the outcomes (single item scales versus multi-item scales), outcomes (e.g., mental health, sleep problems, and work performance). If the study features relevant graphs, data will be extracted using the WebPlotDigitizer software [30]. In cases where the required information is unobtainable, the study will be excluded from the meta-analysis. The authors will ensure that no sample is double counted by verifying that the sample in a given study does not overlap with other included samples from other studies. If an overlap is identified, the data from the largest sample will be extracted and used in the analysis. In the event of any disagreements in terms of extractions, discussion and further review of the studies will be carried out until consensus is reached.

### Assessment of risk-of-bias

As most of the studies are expected to be non-randomized cohort studies, cross-sectional, and case-control designs, the Mixed Methods Appraisal Tool (MMAT) for systematic mixed study reviews will be used [31]. The Mixed Methods Appraisal Tool evaluates the studies based on two screening questions for mixed methods research and 25 items, sorted into five categories: qualitative, quantitative randomized controlled trials, quantitative non-randomized, quantitative descriptive, and mixed methods. The two independent reviewers will assess the risk of bias independently of each other and resolve any differences in opinion through discussion until consensus is reached. If necessary, a third reviewer will be consulted for a definite determination. Inter-rater reliability will be calculated for inclusion/exclusion, data extracted from primary sources and for the risk-of-bias evaluation, resulting in three overall reliability measures. These will be reported as percentage agreement and Cohen’s kappa. Regarding the latter, *K* ≤ 0.20 is considered poor, 0.21 to 0.40 fair, 0.41 to 0.60 moderate, 0.61–0.80 good, and 0.81 to 1.00 very good, respectively [32].

### Meta-analytical approach

The meta-analysis will be carried out using the Comprehensive Meta-Analysis, version 4 [33]. The odds ratio (OR) with a 95% confidence interval (CI) will be reported as the primary measure of effect size. The initial meta-analysis will examine the association between quick returns and specified outcomes. If studies report several effect sizes based on the same sample (e.g., models with diverse control measures), the average of the combined effect sizes will be calculated. As a primary measure of effect size, the OR with associated 95% CI will be reported. First, a general analysis of the association between quick returns and the four specified main outcomes (health, work performance, social factors, and psychosocial work-related factors) will be conducted. In cases where studies report several effect sizes from the same sample, for example, models with different control measures the unadjusted, or the effect size with fewer adjustments will be used. After the initial analyses has been conducted for the four specified main outcomes, separate analyses will be carried out for different types of quick returns (e.g., evening to day shift, day to night shift, etc.) as well as for different types of outcomes (e.g., health, work performance, social factors, and psychosocial work-related factors) provided that at least two relevant studies are available, as this is the basic requirement for meta-analytic estimations [34]. Depending on the level of measurement, moderator analyses in meta-analyses can be conducted as subgroup analyses or as a meta-regression. The overall magnitude and direction of associations between quick returns and the specified outcomes will be determined using cross-sectional and prospective data.

Moderator analysis will be used to assess and explain sex and age differences in the relationship between quick returns and the specified outcomes as well as the moderating effect of occupational groups (healthcare workers versus not), actual resting time between shifts (continuous variable), instruments utilized (single item scales versus multi-item scales), and sample size on quick return and the specified outcomes.

Heterogeneity of the studies will be assessed using Cochran’s *Q* test, where a significant heterogeneity value rejects the null hypothesis of homogeneity. To obtain an estimate of the relative heterogeneity (the degree of variance which reflects true variance between studies), the *I*^2^ statistic will be calculated, with 25% indicating low heterogeneity, 50% indicating medium heterogeneity, and 75% indicating high heterogeneity, respectively [35].

### Publication bias

To investigate publication bias, we will utilize the trim-and-fill procedure developed by Duval and Tweedie [36]. This procedure examines the funnel plot, where effect sizes are plotted against the inverse of the variance (sample size). A symmetrical funnel plot indicates no publication bias, while an asymmetrical plot usually suggests a lack of small studies with small effects. The trim-and-fill procedure identifies and adjusts for asymmetric outlying studies, providing an adjusted effect size and 95% CI.

## Discussion

The existing literature on quick returns and related outcomes has inconsistencies and ambiguous findings which may stem from differences in study samples and the operationalization of central variables. This creates a gap that limits the current understanding of quick returns and potential outcomes. Consequently, the primary goal of the planned systematic review and meta-analysis is to summarize the evidence on the associations between exposure to quick returns and all the health, work performance, social, and psychosocial work-related outcomes that have been examined in the existing literature. The second objective is to gather information on the relationship between different types of quick returns and the specified outcomes, as well as the influencing factors that may moderate these associations. This will be the first meta-analysis to investigate how quick returns impact relevant aspects of workers’ lives such as health, job performance, social factors, and psychosocial work-related factors. Following the recommendations of including ten studies per covariate when investigating potential moderators using meta-regression analyses [37], the number of variables tested in the meta-regression will be restricted. Therefore, depending on the number of studies included in the synthesis, the variables to be tested as potential moderators will be prioritized in the following order: i) age, ii) sex, iii) occupational groups (healthcare workers versus not), iv) instruments utilized to measure the outcomes (single item scales versus multi-item scales), and v) sample size. The insights gained from this study of the existing literature could be beneficial in creating healthy work schedules that promote well-being and positive work conditions, ultimately reducing or minimizing negative outcomes.

In terms of limitations, it is important to acknowledge that the search strategy (i.e., the search strings) will be developed and applied in English and the Scandinavian languages (Danish, Norwegian, and Swedish), which may reduce the retrieval of relevant studies published in other languages, especially those with non-English abstracts. However, full-text articles published in European languages will be considered for inclusion. It is also worthwhile to mention that the studies examined may be heterogeneous with respect to the populations and types of quick returns and the specified outcomes, which may limit the comparability and interpretation of the results.

Additionally, the planned review will incorporate gray literature to ensure a comprehensive overview of the available evidence to minimize publication bias. Gray literature, such as government reports and technical documents, can offer valuable insights that are not always available in peer-reviewed publications. However, a limitation of gray literature is the lack of standardized peer-review processes, which may affect the reliability and quality of the findings. To address this concern, we will systematically assess the risk of bias using an established tool [31]. This approach will facilitate a critical evaluation of the findings for methodological quality and potential biases, thereby providing a balanced and reliable synthesis of the evidence.

## Supplementary Information

Additional file 1: PRISMA-P checklist.

## Data Availability

Not applicable.
